# Supplementary material for: Nanoemulsions as a Promising Carrier for Topical Delivery of Etodolac: Formulation Development and Characterization
Source: Pharmaceutics. 2023 Oct 23;15(10):2510. doi: 10.3390/pharmaceutics15102510 (PMC10610052; doi:10.3390/pharmaceutics15102510)
Supplement: Supplementary file 1 [file pharmaceutics-15-02510-s001.zip › pharmaceutics-2632581-supplementary.pdf]

Nanoemulsions as a Promising Carrier for Topical Delivery of Etodolac: Formulation Development and Characterization

Samet Özdemir [1,*], Burcu Üner [2], Alptuğ Karaküçük [3], Burak Çelik [4], Engin Sümer [5] and Çetin Taş [6]

[1] Department of Pharmaceutical Technology, Faculty of Pharmacy, Istanbul Health and Technology University, 34445 Istanbul, Turkey
[2] Department of Administrative and Pharmaceutical Sciences, University of Health Science and Pharmacy in St. Louis, St. Louis, MO 63110, USA; burcu.uner@uhsp.edu
[3] Department of Pharmaceutical Technology, Faculty of Pharmacy, Ankara Medipol University, 06050 Ankara, Turkey; alptug.karakucuk@ankaramedipol.edu.tr
[4] Department of Pharmaceutical Technology, Faculty of Pharmacy, Bezmialem Vakif University, 34093 Istanbul, Turkey; bcelik@bezmialem.edu.tr
[5] Experimental Research Center (YUDETAM), Faculty of Medicine, Yeditepe University, 34755 Istanbul, Turkey; engin.sumer@yeditepe.edu.tr
[6] Department of Pharmaceutical Technology, Faculty of Pharmacy, Yeditepe University, 34755 Istanbul, Turkey; cetin.tas@yeditepe.edu.tr
* Correspondence: samet.ozdemir@istun.edu.tr.

Selection of Excipients

A previously established method was modified to select the most appropriate oil type for the preparation of nanoemulsions [1]. Caprylic/Capric Triglyceride (Crodamol™ GTCC), Isopropyl Isostearate (Crodamol™ IPIS), Isopropyl Myristate (Crodamol™ IPM), Isopropyl Palmitate (Crodamol™ IPP), Castor oil, Olive oil, and Sesame oil were screened for the determination of highest solubilizing capacity for etodolac. For this purpose, 1 mL of each oil was transferred into 5 mL screw capped glass vials. Subsequently, increasing amounts of etodolac (10 – 250 mg) were added to the vials. 10 mg of addition was applied for each time. The mixtures were then placed in an isothermal shaker (Wise Bath, Daihan Scientific Co Ltd, Korea) at 65 °C, then allowed to equilibrate for 24 hours. Following each addition, a visual inspection was conducted at room temperature to determine the presence of drug crystals. The results are shown in Table S1.

Table S1. Oil screening results.

Added ETD Amount (mg)	Oils						
	CCTG	**IPIS**	IPM	IPP	CO	OO	SO
10	-	**-**	-	-	-	-	-
20	-	**-**	-	-	-	-	-
30	-	**-**	-	-	-	-	-
40	-	**-**	-	-	-	-	-
50	-	**-**	-	-	-	-	+
60	-	**-**	-	-	-	-	
70	-	**-**	-	-	-	-	
80	-	**-**	-	-	-	-	
90	-	**-**	-	-	-	-	
100	-	**-**	-	-	-	-	
110	-	**-**	-	-	-	-	
120	-	**-**	-	-	-	-	
130	-	**-**	-	-	-	-	
140	-	**-**	-	-	-	-	
150	-	**-**	-	-	-	-	
160	-	**-**	-	-	-	-	
170	-	**-**	+	-	+	+	
180	-	**-**		-			
190	-	**-**		-			
200	-	**-**		-			
210	+	**-**		+			
220		**-**					
230		**-**					
240		**-**					
250		**+**					

ETD: Etodolac, CCTG: Caprylic/Capric Triglyceride, IPIS: Isopropyl isostearate, IPM: Isopropyl myristate, IPP: Isopropyl palmitate, CO: Castor oil, OO: Olive oil, SO: Sesame oil. Positive symbol (+) indicates the presence of drug crystals.

Surfactant type was determined using a method previously reported [2]. In brief, coarse emulsions of the selected oil were prepared using high shear homogenization. A midpoint oil concentration of 5% (w/w) was chosen as the oil phase, and a low concentration of surfactant (1%, w/w) containing the aqueous phase was prepared. Both phases were mixed using a high shear homogenizer (Silent Crusher M, Heidolph, Germany) at 25000 rpm for 1 minute. The homogenization process was conducted at a thermal condition of 65°C. The coarse emulsions were left in a dark place overnight at room temperature and were subsequently visually inspected. The observations are shown in Table S2.

Table S2. Surfactant screening results.

Surfactant	Observation
Poloxamer® 188	Phase separation
Poloxamer® 407	Phase separation
Brij® 35	Phase separation
**Tego Care® 450**	**Non-viscous (liquid)**
Tyloxapol	High-viscous (semisolid)

Optimization of Formulations

Table S3. Coefficients of placebo formulation variables according to initial design

	DS			PDI			ZP		
Source	F-value	p-value	R^2	F-value	p-value	R^2	F-value	p-value	R^2
Model	0.938	0.471*		2.21	0.482*				
Linear Mixture	2.42	0.413*		2.22	0.429*		0.784	0.424*	
AB	0.00596	0.451*		1.08	0.488*		0.528	0.400*	
AC	0.0982	0.307*		0.502	0.308*		0.987	0.302*	
BC	0.409	0.338*		0.0774	0.427*		0.788	0.338*	
ABC	0.000187	0.991		1.06	0.490*		0.682	0.561	
AB(A-B)	0.263	0.698		0.0149	0.923		1.16	0.477*	
AC(A-C)	0.049	0.841		0.445	0.626		1.05	0.492*	
BC(B-C)	0.595	0.582		0.0330	0.886		0.0276	0.895	
			0.988			0.954			0.976

A: surfactant, B: oil, C: distilled water, * indicates significance (p<0.05)

Table S4. Coefficients of medicated formulation variables according to secondary design

	DS			PDI			ZP			EE		
Source	F-value	p-value	R^2	F-value	p-value	R^2	F-value	p-value	R^2	F-value	p-value	R^2
Model	68.1	0.0931*		10.4	0.0903*		169.	0.0591*		0.118	0.377*	
Linear Mixture	146.	0.0484*		7.60	0.116*		412.	0.0348*		0.153	0.375*	
AB	16.4	0.154*		25.9	0.0365*		148.	0.0523*		0.000451	0.486*	
AC	5.83	0.250*		33.0	0.0290*		208.	0.0441*		0.00217	0.570	
BC	95.9	0.064*		3.74	0.193*		8.51	0.210*		0.00309	0.465*	
ABC	8.33	0.212*		28.7	0.0331*		182.	0.0471*		0.000655	0.584	
AB(A-B)	8.11	0.215*		8.04	0.216*		123.	0.0572*		0.00392	0.560	
			0.998			0.969			0.999			0.808

A: surfactant, B: oil, C: distilled water, * indicates significance (p<0.05)

Quantification of Etodolac

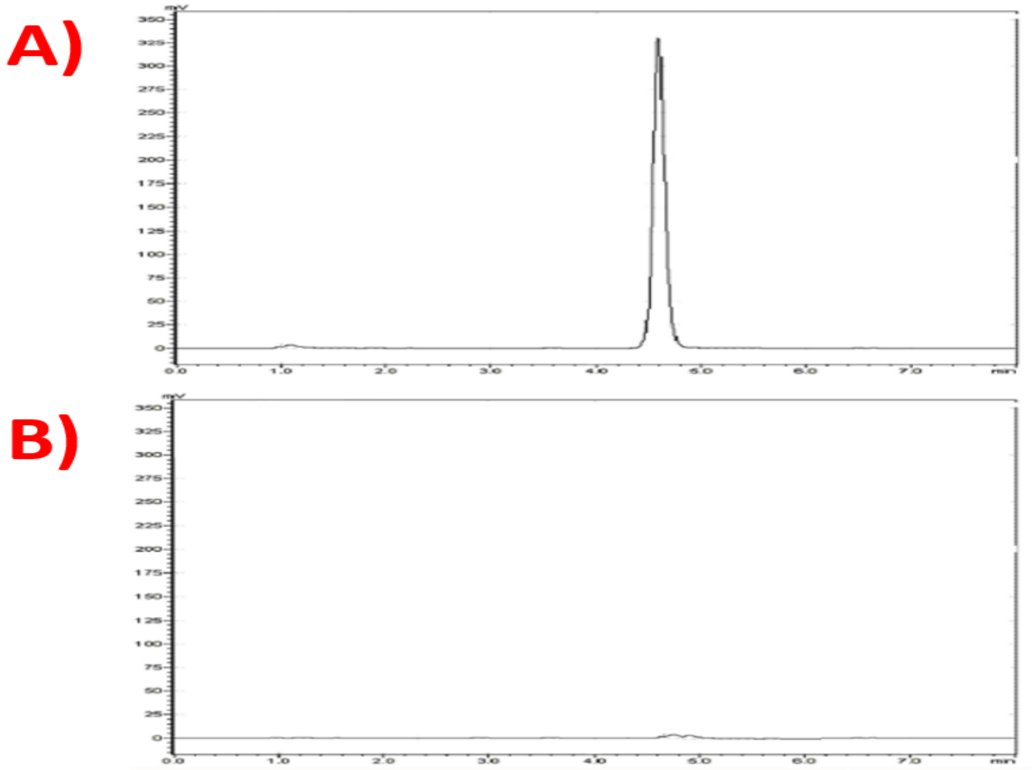

Figure S1. HPLC chromatograms A: etodolac peak and B: mobile phase peak

Tape Stripping Study

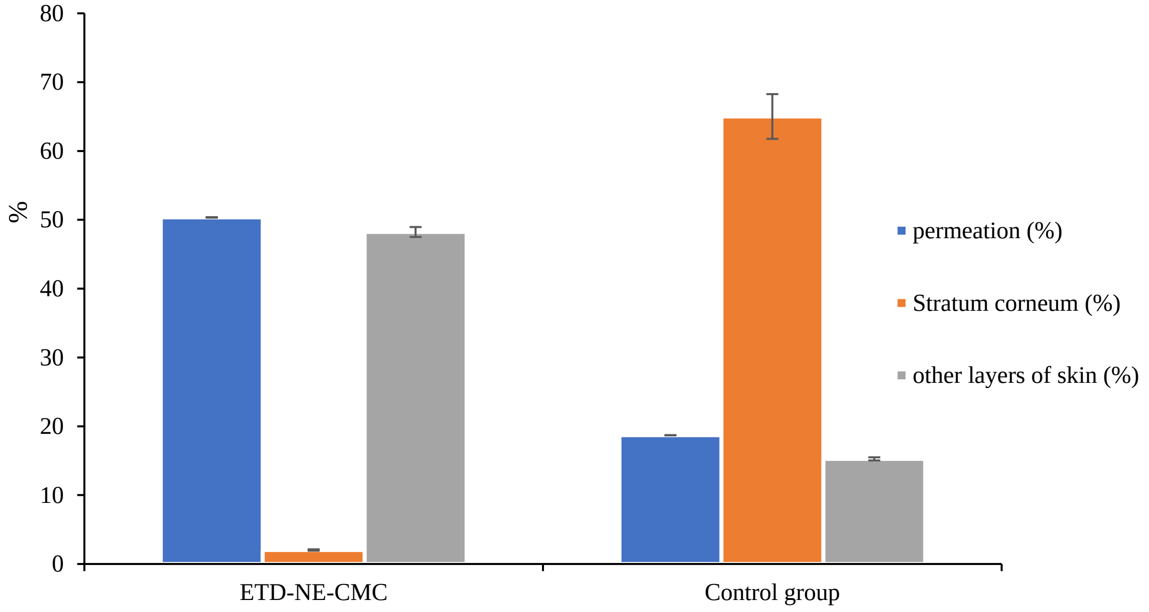

Figure S2. Skin presence of etodolac after *ex vivo* permeation study

Stability Study

Table S5. Monitorization of physical stability of optimal nanoemulsion (* indicate significant difference, p<0.05)

25 ° C						
**Day**	**DS (nm)**	**PDI**	**ZP (mV)**	**EE (%)**	**pH**	**Viscosity (cP)**
0	165.50 ± 0.9	0.123 ± 0.014	-34.28 ± 1.12	92.69 ± 1.72	6.8 ± 1.1	15.69 ± 2.15
1	165.40 ± 1.0	0.234 ± 0.019	-34.35 ± 0.62	90.09 ± 1.99	6.8 ± 1.0	16.25 ± 1.21
3	167.80 ± 0.7	0.21 ± 0.019	-34.42 ± 1.65	87.48 ± 1.21	6.7 ± 0.9	15.97 ± 1.43
7	173.80 ± 0.9	0.199 ± 0.021	-34.49 ± 1.98	87.87 ± 1.40	6.7 ± 0.5	17.01 ± 1.98
30	174.03 ± 0.5	0.201 ± 0.021	-34.56 ± 2.05	85.26 ± 1.55	6.8 ± 0.3	17.23 ± 1.93
60	175.50 ± 0.8	0.23 ± 0.020	-34.63 ± 1.13	81.65 ± 1.19	6.7 ± 0.1	17.17 ± 1.05
90	176.00 ± 1.1	0.197 ± 0.022	-34.70 ± 3.62	80.09 ± 0.56	6.6 ± 0.3	17.22 ± 1.09
**40 ° C**						
**Day**	**DS (nm)**	**PDI**	**ZP (mV)**	**EE (%)**	**pH**	**Viscosity (cP)**
0	167.4 ± 1.3	0.188 ± 0.027	-32.72 ± 1.19	92.69 ± 1.40	6.8 ± 1.7	16.25 ± 0.96
1	178.2 ± 2.1	0.193 ± 0.012	-33.62 ± 1.56	89.05 ± 1.78	6.7 ± 0.9	15.25 ± 1.19
3	177.8 ± 2.9	0.198 ± 0.040	-34.28 ± 1.72	85.41 ± 1.03	6.7 ± 0.3	15.59 ± 0.65
7	182.5 ± 1.6	0.203 ± 0.005	-35.10 ± 1.98	81.76 ± 1.72	6.6 ± 0.4	14.55 ± 0.76
30	193.9 ± 1.4*	0.208 ± 0.013	-35.88 ± 0.66	78.12 ± 1.86	6.7 ± 0.3	14.25 ± 0.49*
60	203.3 ± 1.2*	0.213 ± 0.019	-36.66 ± 1.19	74.47 ± 1.99*	6.1 ± 0.8	14.15 ± 0.41*
90	214.9 ± 0.5*	0.218 ± 0.045	-37.44 ± 1.40	70.83 ± 1.59*	5.9 ± 0.8*	14.11 ± 0.17*

DS: droplet size, PDI: polydispersity index, ZP: Zeta potential, EE: encapsulation efficiency.

References

1. Cirri, M.; Maestrini, L.; Maestrelli, F.; Mennini, N.; Mura, P.; Ghelardini, C.; Di Cesare Mannelli, L. Design, Characterization and in Vivo Evaluation of Nanostructured Lipid Carriers (NLC) as a New Drug Delivery System for Hydrochlorothiazide Oral Administration in Pediatric Therapy. *Drug Deliv.* **2018**, *25*, 1910–1921, doi:10.1080/10717544.2018.1529209.
2. Özdemir, S.; Çelik, B.; Türköz Acar, E.; Duman, G.; Üner, M. Eplerenone Nanoemulsions for Treatment of Hypertension. Part I: Experimental Design for Optimization of Formulations and Physical Characterization. *J. Drug Deliv. Sci. Technol.* **2018**, *45*, 357–366, doi:https://doi.org/10.1016/j.jddst.2018.03.011.
